# Trypan Blue Dye Enters Viable Cells Incubated with the Pore-Forming Toxin HlyII of *Bacillus cereus*

**DOI:** 10.1371/journal.pone.0022876

**Published:** 2011-09-06

**Authors:** Seav-Ly Tran, Andrea Puhar, Maud Ngo-Camus, Nalini Ramarao

**Affiliations:** 1 INRA, Unité MICALIS, UMR 1319, Guyancourt, France; 2 Unité PMM, INSERM U786, Institut Pasteur, Paris, France; Universite de la Mediterranee, France

## Abstract

Trypan blue is a dye that has been widely used for selective staining of dead tissues or cells. Here, we show that the pore-forming toxin HlyII of *Bacillus cereus* allows trypan blue staining of macrophage cells, despite the cells remaining viable and metabolically active. These findings suggest that the dye enters viable cells through the pores. To our knowledge, this is the first demonstration that trypan blue may enter viable cells. Consequently, the use of trypan blue staining as a marker of vital status should be interpreted with caution. The blue coloration does not necessarily indicate cell lysis, but may rather indicate pore formation in the cell membranes and more generally increased membrane permeability.

## Introduction

Trypan blue is a diazo dye that has been widely used to color dead tissues or cells selectively. The mechanism of trypan blue staining is based on it being negatively charged and not interacting with cells unless the membrane is damaged. Indeed, undamaged cells are very selective concerning the compounds that pass through their membrane, and thus should not take up trypan blue. Therefore, all the cells that exclude the dye are considered viable. By contrast, cells with damaged membranes are stained in a distinctive blue color readily observed under a microscope. Thus, trypan blue dye is described as being a vital stain allowing discrimination between viable cells and cells with damaged membranes that are usually considered to be dead cells.

Several bacterial pore-forming toxins can cause host cell damage by perforating the host cell membranes [1], [2], [3]. It is conceivable that the pores formed by the toxin may allow trypan blue to enter the host cell, which would consequently be considered to be dead. Indeed, the trypan blue is a relatively small molecule (960 Da) and may therefore be able to penetrate into cells through pores formed by toxins. However, the issue of whether pore-forming toxins may cause membrane leakage without inducing immediate cell death has not been extensively considered. In this case, trypan blue staining would reveal pore formation in the host cell membrane but not necessarily host cell death.

*Bacillus cereus* is a Gram-positive human food-poisoning pathogen responsible for gastroenteritis [4], and for other serious local and systemic infections [5]. *B. cereus* pathogenicity is multifactorial and likely depends on the production of a high variety of toxins (ie: haemolysins, proteases, phospholipases, non protein-toxins…) [4], [6], [7], [8], [9], [10], [11], [12], [13], [14]. Among these toxins, *B. cereus* expresses an Haemolysin, HlyII, which induces pore formation in various mammalian cells, including macrophages and erythrocytes [15], [16]. The diameter of the pores formed ranges from 1.5 to 4.6 nm [17]. At high doses, HlyII causes pore formation eventually leading to death by apoptosis in human and mouse monocytes and macrophages [18]. Here, we show that HlyII allows trypan blue staining of the cells. At low HlyII doses, the pore formation is transient and cells can subsequently recover. Indeed, the cells remain viable and display metabolic activity, strongly suggesting that the dye enters viable cells through the pores.

## Results and Discussion

**Figure 1:**
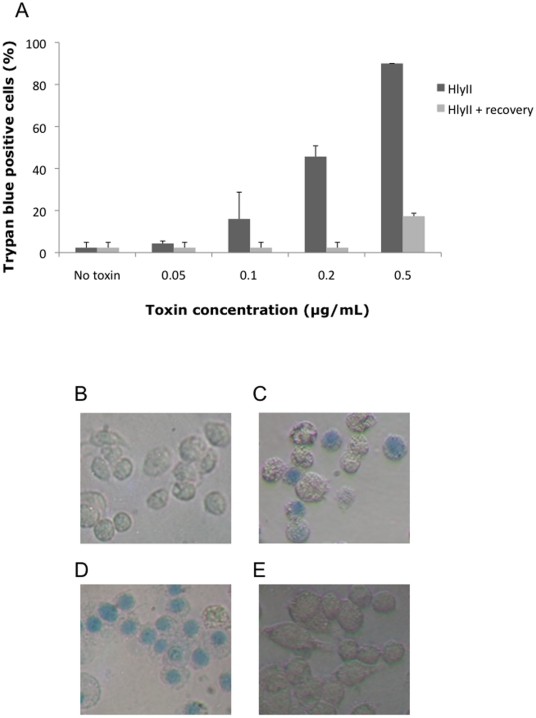
HlyII induces transient membrane permeability. Macrophages were incubated with increasing concentrations of HlyII (0 to 0.5 µg/mL) for 2 h, and membrane permeability was assessed by trypan blue dye exclusion (black scale). A representative image is shown for non-treated cells (B), and cells incubated with HlyII at 0.2 µg/mL (C) and 0.5 µg/mL (D). Alternatively, macrophages were incubated with HlyII (0 to 0.5 µg/mL) for 2 h, and washed to remove the toxin. The macrophages were then allowed to recover in fresh medium supplemented with FBS for 24 h. After recovering, membrane permeability was assessed by trypan blue dye exclusion (grey scale). A representative image is shown of recovered cells after incubation with HlyII at 0.2 µg/mL and 24 h recovery (E).

To study the effect of HlyII on host cell membrane integrity, murine J774 macrophages were incubated with various doses of purified GST-tagged HlyII (0–0.5 µg/mL) as previously described [18]. After 2 h of incubation, trypan blue dye was added to the preparation and cells were visualized under the microscope. The percentage of total cells that was blue was calculated by counting, for each condition, 100 cells from three independent experiments (Fig. 1A, dark columns). HlyII at a concentration of 0.2 µg/mL resulted in 50% of the cells to be stained blue (Fig. 1A, 1C), and at 0.5 µg/mL nearly 100% of the cells were stained blue (Fig. 1A, 1D). HlyII has been described as a pore-forming toxin [17], [19]. We have previously shown that HlyII induces macrophage apoptosis after 24 h incubation [18] and this seemed in apparent contradiction with a trypan blue staining as indicator of cell death after 2 h of incubation. Indeed, if the trypan blue dye staining reflects cell death as usually described, the cells should be already dead after 2 h of incubation with the toxin, and therefore be unable to undergo apoptosis subsequently. Therefore, we hypothesized that the trypan blue dye, which is a small molecule, may enter the cells through the pores formed by HlyII. To investigate this issue, we used various techniques to test whether the trypan blue staining detected within 2 h of incubation with HlyII was a consequence of pore formation rather than immediate cell necrosis.

We first assessed whether the membrane permeabilization revealed by trypan blue staining was reversible, that is whether the infected cells could recover and generate new membrane after elimination of the pore-forming toxin. Cells were incubated with various concentrations of HlyII for 2 h, and membrane permeabilization was monitored by trypan blue staining (Fig. 1A, dark columns). Alternatively, after 2 h incubation with HlyII, the toxin was removed by intensive washing and cells were allowed to recover in new RPMI medium supplemented with 10% FBS (foetal bovine serum, Sigma) for 24 h. Membrane permeabilization was then assessed by trypan blue staining (Fig. 1A, light columns). After the 24 h incubation in fresh medium (the recovery period), less than 10% of the cells incubated with 0.2 µg/mL HlyII (Fig. 1A, 1E) were still blue (to be compared to 50% immediately after 2 h incubation with 0.2 µg/mL HlyII without a recovery period, Fig. 1A, 1C). Thus, the cell membrane was no longer permeable to the dye. When cells were incubated with 0.5 µg/mL of HlyII, the proportion of blue cells dropped from 90% after 2 h of incubation to 17% after the recovery period. Thereafter, HlyII-treated cells were capable of recovering from membrane permeabilization and of reforming intact membranes. This shows that the cells were still viable after 2 h incubation with various concentrations of HlyII.

**Figure 2:**
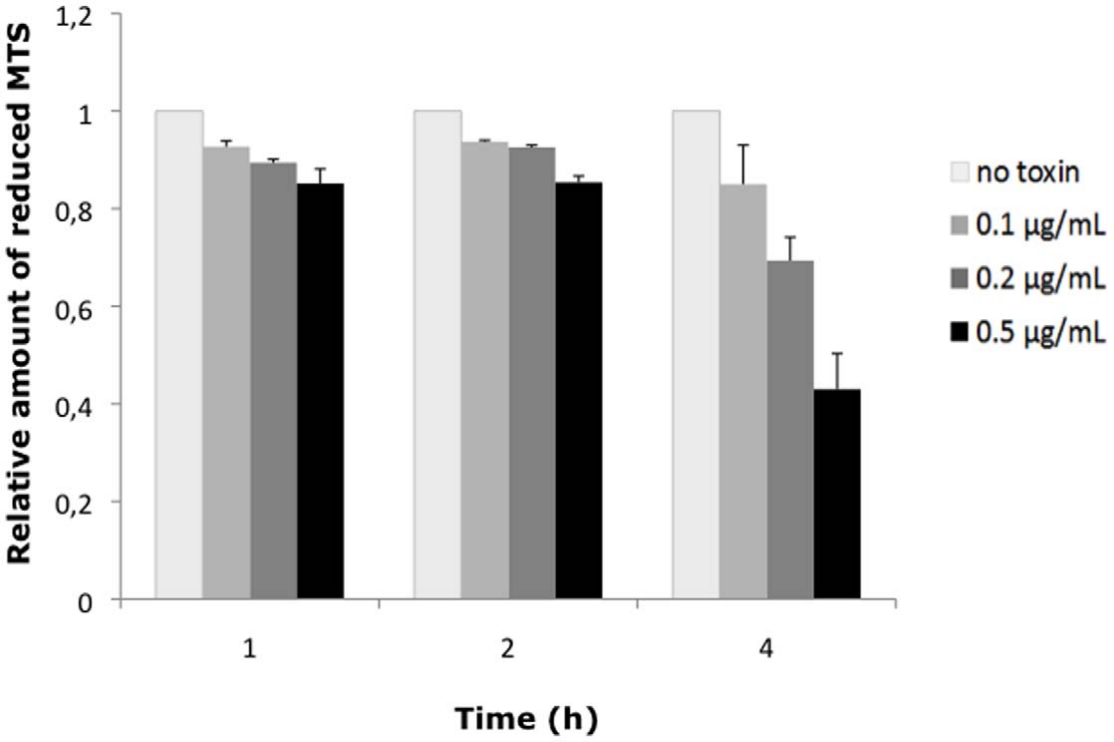
Cells have normal metabolic activity following incubation with HlyII. Macrophages were incubated with increasing concentrations of HlyII (0 to 0.5 µg/mL) for the times indicated. Cellular metabolic activity was evaluated by measuring absorbance at 490 nm, which is proportional to NADH- or NADPH-dependent reduction of 3-(4,5-dimethylthiazol-2-yl)-5-(3-carboxymethoxyphenyl)-2-(4-sulfophenyl)-2H-tetrazolium) (MTS) for up to 4 h after incubation with the toxin. The values were normalized to those for untreated cells (no toxin).

To confirm the viability of the cells incubated for 2 h with HlyII, their metabolic activity was assessed by measuring the NADH or NADPH-dependent reduction of 3-(4,5-dimethylthiazol-2-yl)-5-(3-carboxymethoxyphenyl)-2-(4-sulfophenyl)-2H-tetrazolium) (MTS). The CellTiter96 Aqueous One Solution Cell Proliferation Assay (Promega) was used at the indicated times after treatment with 0–0.5 µg/ml of HlyII toxin, and absorbance was read in a Tecan Sunrise microplate reader. Cells incubated with up to 0.5 µg/mL HlyII showed metabolic activity comparable to that of untreated cells, for up to 2 h after incubation (Fig. 2). After 4 h incubation, the metabolic activity of HlyII-treated cells decreased and dropped to 70% of control values when incubated with 0.2 µg/mL HlyII and to 40% with 0.5 µg/mL HlyII. Thus, despite the blue coloration by trypan blue, the cells retain their metabolic capacity for up to 2 h in the presence of HlyII, and are therefore viable. Only after 4 h of incubation with HlyII did the cell metabolic activity decrease substantially. This is consistent with our previous finding that HlyII induces apoptotic signals that can be measured by Annexin V staining and activation of caspases after 4 h of incubation [18].

**Figure 3:**
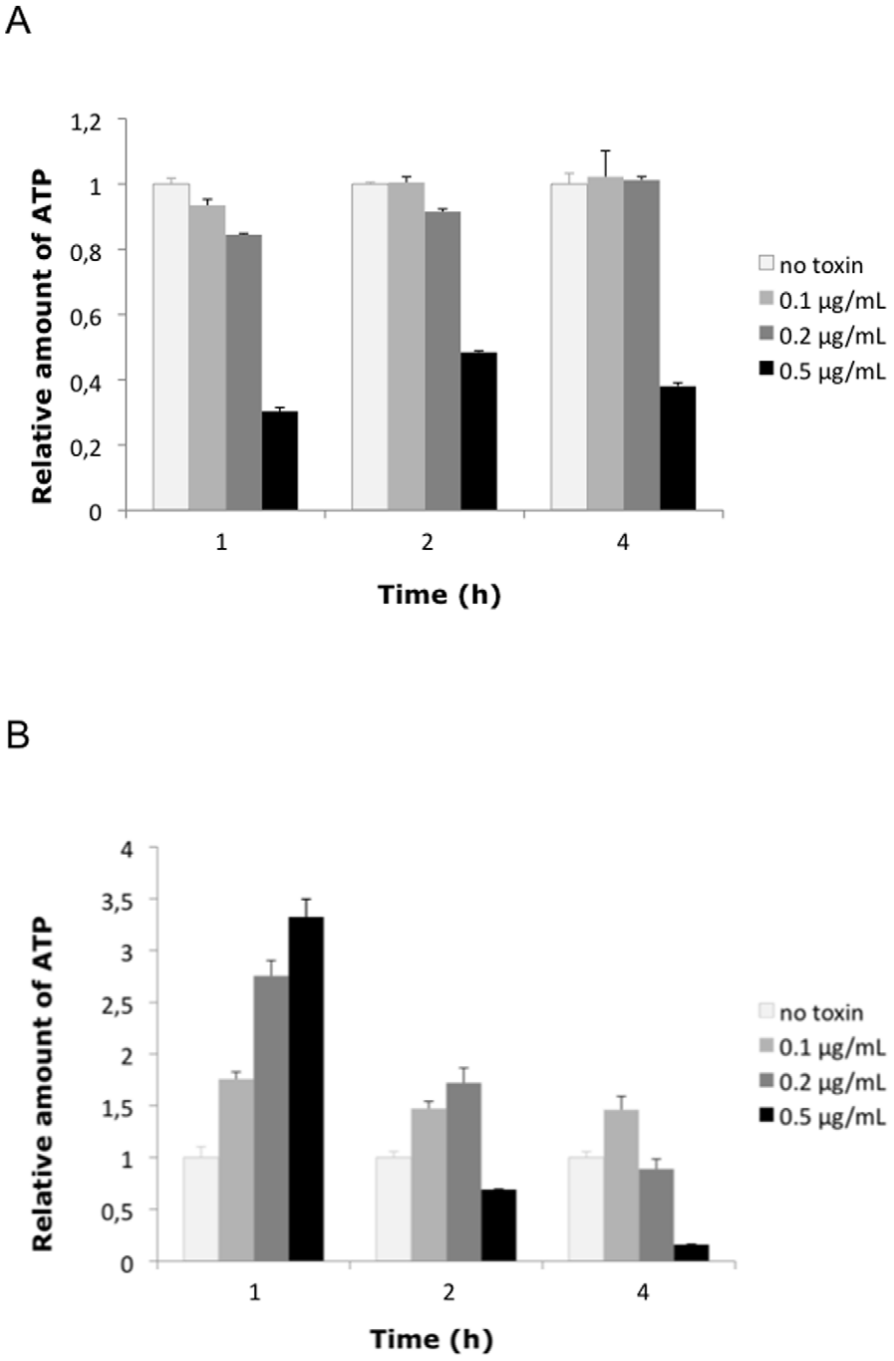
ATP production following incubation with HlyII. Macrophages were incubated with increasing concentrations of HlyII (0 to 0.5 µg/mL) for the times indicated. Intracellular (cell lysate) (A) and extracellular (supernatant) (B) ATP concentrations were determined. The values reported for ATP concentration have been normalized to those for untreated cells (no toxin).

To confirm the viability of the cells despite membrane permeabilization, we monitored cellular ATP production (Fig. 3). J744 macrophages were treated with 0–0.5 µg/mL of HlyII for the indicated times. The culture supernatants were collected and the cells were lysed. The ATP contents of supernatants (extracellular) and cell lysates (intracellular) were assessed using an ATP Determination Kit (sensitive assay, Proteinkinase.de) according to the manufacturer's instructions. Cells incubated with 0.1 µg/mL of HlyII showed intracellular ATP concentrations similar to those in untreated cells (Fig. 3A). Incubation with 0.2 µg/mL of toxin for 1 or 2 h did not substantially change the intracellular ATP concentration. In cells treated with 0.5 µg/mL of toxin, the ATP concentration decreased to around 30% after 1 h incubation and increased to 50% after 2 h of incubation compared to untreated cells. After 4 h of incubation the ATP concentration in cells treated with 0.1 µg/mL or 0.2 µg/mL HlyII had levels similar to those in untreated cells. By contrast, the intracellular ATP concentration in cells incubated with 0.5 µg/mL HlyII continued to decline, indicating that, at a high toxin concentration, cells are too damaged to regenerate the lost ATP. At early time points, incubation with the toxin caused a dose-dependent release of the ATP from the cells (Fig. 3B), probably through the pores formed by HlyII; presumably, the ATP diffused along its concentration gradient from the cytosol (1–3 mM in healthy cells) to the extracellular medium, where ATP is scarce. Indeed, after 1 h of incubation with 0.5 µg/mL of toxin, the concentration of extracellular ATP was over three fold higher than that for untreated cells. At longer incubation times, the amount of extracellular ATP decreased. In cultures treated with 0.1 µg/mL or 0.2 µg/mL HlyII, the extracellular ATP concentration fell to only slightly higher than that in untreated cultures. This may reflect the regeneration of damaged membranes and consequently reduced ATP leakage. However, in cultures treated with 0.5 µg/mL HlyII, extracellular ATP concentration dropped to below values for untreated cultures. This may have been a consequence of the substantial decline in intracellular ATP production. These findings show that cells incubated with HlyII were viable and capable of producing ATP, although significant numbers of them were permeable to the trypan blue dye.

Taken together, these findings clearly show that the membrane permeabilization revealed by trypan blue dye staining is essentially the result of the pore formation by HlyII. This pore formation does not induce immediate death of the cells, which are still able to produce ATP, are metabolically active and can repair their damaged membrane. Nevertheless, the formation of the pores subsequently provoke cell suicide. Indeed, apoptotic markers are detectable after 4 h of incubation with the toxin [18].

Despite the obvious consequences of membrane perforation, cells have evolved mechanisms that allow them to recover their plasma membrane integrity, given that the extent and duration of damage is not overwhelming [20], [21]. Previous studies have reported that pores caused by the bacterial toxin streptolysin O (SLO) can be repaired with the same rapid kinetics observed for the resealing of mechanical wounds, by removal or disassembly of the transmembrane pores [22]. As for HlyII, repair occurs spontaneously in the presence of physiological media. A drop of the intracellular ATP level followed by a rise to level of control cells, accompanies cell membrane damage and recovery, respectively. Similarly, for the staphylococcal α-toxin, the small transmembrane β-barrel can be constricted, resulting in pore closure and cell recovery [23]. Transient breakdown of the membrane permeability barrier followed by cell recovery is likely relevant in the context of infection, immunity and tissue regeneration.

Our experiments show that pore-forming toxins, as exemplified by HlyII, can induce pore formation in host cell membranes leading to membrane permeabilization, measurable by trypan blue staining, without causing immediate cell death. To our knowledge, this is the first demonstration that the trypan blue may enter viable cells, although it has already been suggested in various models [23], [24], [25]. The use of trypan blue as a vital dye should therefore be considered with caution. Blue coloration of cells may not necessarily indicate cell lysis, but may rather reveal the formation of pores in the cell membranes and more generally membrane permeability. In any case, cell death will occur, if the flow through the cell membrane reaches a lethal extent.
